# Supplementary material for: Perceiving depth beyond sight: Evaluating intrinsic and learned cues via a proof of concept sensory substitution method in the visually impaired and sighted
Source: PLoS One. 2024 Sep 25;19(9):e0310033. doi: 10.1371/journal.pone.0310033 (PMC11423994; doi:10.1371/journal.pone.0310033)
Supplement: Attachment — Submitted filename: PLOS ONE Replies to Reviewers.docx [file pone.0310033.s001.docx]

Dear Editor,

Thank you for providing us with the reviewers' useful comments and for allowing us to revise the manuscript accordingly. As requested, we have now carried out a major revision of the manuscript as detailed below.

Let me focus on some points that the reviewers and I would like to see addressed. These are:

1) Important details and background are missing for the auditory parameters used.

2) Overly long writing and several paragraphs with irrelevant information.

3) The frequent use of the term “ecological” needs clarification, as it does not seem fully justified sometimes.

4) Minor points from me:

Line 111: “Bouba Kiki” is the usual name;

Line 216: “the x and z”: should not be “the y and z”?

Line 220: “and Test - in which participants”...

Line 266 and elsewhere: “Cohen’s d” with capital C

Lines 266 and 267: please revise the punctuation marks

Line 272: Intechanged

There are other points brought out in the reviews and I will carefully attend to your item-by-item responses to them.

- We have now addressed these major points and corrected all of the minor points. Mainly, we focused a lot of the writing, removing less relevant segments and replaced them with the requested content, including the auditory parameters and an better defined and consistent use of the ecological terminology. We detail below our item-by-item responses to the other points brought out in the reviews, and indicate how and where we addressed them by tracking the changes in the manuscript document.

# Reviewer #1:

The paper reports the behavioral testing of a minimalist sensory substitution method incorporating spoken words that are manipulated using a low-pass filter and pitch to indicate the target locations distance and verticality respectively. This method is compared against an alternative method that swaps the distance and verticality mappings. They find initial divergence in scores (with the initial method being superior) which convergences to equivalent performance after training in the test condition. Overall, the experiment and its findings are fairly straight forward, which should lend itself to a leaner manuscript. Furthermore, important details and background are missing for the auditory parameters used.

The general impression I get is that the writing is frequently overly long which tends to obscure the actual meaning that is attempted to be conveyed. It feels like sentences frequently tend to meander slightly on their way to the point, rather than writing for clarity and efficiency. You could probably eliminate about 30% of the words in this manuscript and have no loss in the actual information provided. By being more exact in the language used, it should make the paper more readable, intuitive, and give yourself space to add crucial information which is missing (e.g. the exact audio used by the system). On top of this, not all writing is relevant enough to be included, so a couple of paragraphs could be cut as well - when evaluating a paragraph ask whether this information directly relates to the experiment, or is this writing for writing's sake?

Finally, there is a bit of overselling at the end about spatial skills being inferred from this experiment, but the experiment itself is an abstract task involving 9 spatially organised buttons. The users are not meaningfully exploring and interacting with an ecologically valid 3D space, so it could be argued that this is closer to an auditory discrimination and memory task, than a distance perception task. This is fine as an experiment, but I think the overall insights that can be gained from this need to be toned down a little as a result.

Below I have comments for each section of the manuscript, I hope the authors find this useful.

- Thank you for these helpful comments. We have now addressed them one by one as detailed below, and as tracked changes in the document file.

## Abstract

“ecologically inspired auditory properties” - ecologically tends to mean ‘in the environment’ so would need justification or just that it's a psychological intuition (shown by cross-modal correspondence research). Perhaps a description of the auditory properties is suitable here.

- When we use the term ecologically, we mean inspired by the environment, as the algorithm employs auditory effects based on those occurring in nature.
  We have added a description of the auditory properties as suggested at the end of the abstract when explaining the conditions, and further detail the exact manipulation performed as requested in the Methods section. Following this and the other comments on the subject, we also made sure to refer to the mapping as "ecologically inspired" throughout the paper, and explained in what way and to what extent it relies on effects occurring in nature.

Give actual numbers of sighted, visually-impaired, and blind participants

- We have now added the exact numbers of sighted, visually-impaired, and blind participants.

“Inherentness” is a vague term to be discussed for testing, please be more exact. What effect are you looking for - reaction time, accuracy, participant ratings? It appears to be initial performance results and post-training results. I’m split on whether inherent is the best word choice, perhaps Intrinsic, implicit, initial, baseline, or intuitive might be better choices.

- Thank you for these suggestions, we have chosen to replace the word *inherently* with *intrinsic* that we agree would make a more appropriate choice.

Some of the language is overly long and vague, where more concise specific language would aid readability.

- We have re-structured the abstract, removed vague phrasings and replaced them with more straightforward explanations based on the suggestions.

“They also indicate that with training the spatial abilities of the visually impaired and blind can converge with those of the sighted.” Be clear this is talking about the SSD performance.

- Thank you for pointing this out, we agree the previous phrasing was broad and not sufficiently clear. Our intention here as we now clarify in the text was to point out that our findings are also relevant in supporting the convergence view as further expanded upon in the Discussion section.

## Introduction

Please add reference for sensory substitution definition.

- We have added references as requested.

“The first involves a conversion of full images to audio or tactile” stimulation.

- We have corrected this.

Line 85 onwards - Would be good to explicitly state how spatial information is conveyed in the topospeech approach.

- We have now added the detailed information as requested in the methods section, and direct to it in the introduction.

There is a lot of dancing around saying the exact thing that is being done “The implementation proposed above, using ecological representation of depth through auditory properties” when you can just say the auditory properties being used. This would help the reader understand and conceptualise what it is like to experience this sensory stimulation.

- We have now simplified this section to clearly explain the conditions and auditory properties being used as suggested.

“representation of the two axes” which two axes? Are you referring to an inverted correspondence between distance and the proposed ecologically valid auditory property? Or applying these to different axes? In 3D perception, intuitively there are 3 axes, horizontal, vertical, depth. So when two are described, which two?

- We have now indicated clearly that we refer to the vertical and depth axes, as well as described the representation used for both.

Looking further into the paper, while it appears distance is being focused on, isn’t verticality being focused on as well? If those are the two axes, they should be explicitly stated. E.g. line 92 “This work expands upon our previous implementation that represented objects and their horizontal and vertical locations, by representing depth.” [and vertical locations]. My understanding at this point is simply that unlike the prior topospeech studies, horizontal localization is the one axis not being explored.

- It is true that the horizontal axis was removed, and indeed there are conclusions to be drawn also regarding the vertical axis. We have worked through the different sections to refer explicitly to verticality as well as distance, and clarify the representation used for the vertical axis. We ensure it is clear that the mapping of the vertical axes was tested before, and the main focus of the current paper is on the depth representation, and what can be learned from the differences between the two representations.

106 - “depth awareness” Depth discrimination as this is probably the most accurate term.

- Thank you, we have corrected this.

108 - I feel you should be more explicit in the initial sentence that these are psychological intuitions and associations being discussed here.

- We have now rephrased this to explicitly state that these are psychological intuitions and associations as recommended.

I find it unusual that word-based synaesthesia is brought up but not abstract forms like music or pitch-related synaesthesias, since you use pitch as one of the axes later on.

- We have now brought up both pitch and music related synaesthesia as suggested.

122 - “moreare”

- This has been corrected.

The paragraphs on synaesthesia that are not directly related to the experiment could be deleted (lines 125-129). Sticking to visual and audition will help keep the manuscript more concise.

- We have done as recommended and deleted these paragraphs.

It could be argued that pitch-height correspondences can be considered innate from pre-linguistic infants having a looking preference for bouncing balls that rise and fall with congruent pitch changes (see below). There are a variety of studies for infants, and for whether these associations exist blind and low vision populations.

Walker, P., Bremner, J. G., Mason, U., Spring, J., Mattock, K., Slater, A., & Johnson, S. P. (2010). Preverbal infants’ sensitivity to synaesthetic cross-modality correspondences. Psychological Science, 21(1), 21-25.

- This is indeed an excellent, very relevant reference, thank you! We have now referred to this work.

Line 139 - you use the acronym SSD but you haven’t introduced it previously.

- We have now introduced the acronym in the appropriate place.

You could discuss research that shows that SSDs that use cross-modal correspondence have better performance to bolster the idea that you expect this to occur.

- We have included references to prior research on how correspondences can be beneficial for enhancing the performance of sensory substitution users in the Representations of spatial information section.

If you want to discuss how frequency pass filtering can be used for spatialization (specifically verticality), you could look at ‘virtual acoustic space’ papers or the below reference, which uses band-passed noise to indicate vertical positioning (mimicking auditory changes from the ear pinnae - called head-related transfer function). This may have implications for how the swapped condition performs with low-pass filtering is used for verticality.

González-Mora, J. L., Rodriguez-Hernandez, A., Burunat, E., Martin, F., & Castellano, M. A. (2006, April). Seeing the world by hearing: Virtual Acoustic Space (VAS) a new space perception system for blind people. In 2006 2nd International Conference on Information & Communication Technologies (Vol. 1, pp. 837-842). IEEE.

Richardson, M., Thar, J., Alvarez, J., Borchers, J., Ward, J., & Hamilton-Fletcher, G. (2019). How much spatial information is lost in the sensory substitution process? Comparing visual, tactile, and auditory approaches. Perception, 48(11), 1079-1103.

- Thank you for bringing this up. As a proof of concept, we have chosen the audio manipulation to be one that accentuates the differences in a noticeable way, relying on physical effects over distance, rather than the more subtle changes related to the HRTF which is also very individual and more complex to model. We now explain our choices and motivations both in the Methods section when detailing the algorithm, and in the Future directions section where we also now suggest how the algorithm could be further developed with methods such as the ones you have suggested.

Line 141-143 - or no preference would yield the same result

- Very good point. We now refer to this possibility as well. (Note that this was moved to the end of the introduction to consolidate the aims of the study as suggested by Reviewer 2, and for focusing the introduction as requested)

I feel like the introduction overall could be pruned and focused more by asking ‘is this point directly relevant to the study, or is this a tangent?’ and eliminate all the tangents. This would give space for other more relevant information and arguments, such as on pitch-height and pitch-distance associations. Cross-modal correspondences improving user performance on SSDs. Effect of blindness on cross-modal correspondences. Or exploring the auditory characteristics of distance perception (i.e. “the ecological cues”).

- Building on the other comments, we now re-organized and focused this section, while adding further relevant information based on the suggested points

Line 172 - I thought the prior algorithm did not do depth? I presume the algorithm altered or repurposed for this study?

- This is correct, we have now better clarified this in the manuscript.

Ecological is used to refer to the distance parameters and scene richness. But also the experiment is abstracting out a scene and not providing rich visual detail, so this is more of a discussion point than a goal of the present research. No evaluation of verticality is mentioned here. I feel like lines 171-181 could be more ‘to-the-point’.

- We have now focused this section, added the hypotheses to consolidate the research goals as requested by reviewer two, and removed the note on the ecological information, which was indeed not fitting in this context. As mentioned in the previous comments, we added the information regarding verticality and how it's relevant to these goals earlier in the introduction.

## Methods

“Out of the participants, five were blind, and two were visually impaired.” from the information provided the age and gender of the blind and visually-impaired participants cannot be determined.

- We have now added this information.

“Recruitment for the study took place between May 14, 2023 to December 13, 2023” don’t think this information is needed.

- This information was required upon submission of the manuscript, though we have now shortened it to include only the months and years.

“it uses a sweep line technique to scan a visual scene from left to right. It was utilized for conveying spatial information in the x and y axes” If the scanline is not altering the pitches used, then its just utilized for the X axis here.

- This is correct. The sweep line affected the time modulation, representing the location on the horizontal axis. As such, as for your question below, it was not used in this version. We added a clarification of this in the text when describing the new algorithm in the introduction and removed the explanation of the horizontal axis in methods to prevent any confusion, and in line with Reviewer 2's comment on the subject.

“The y-axis location was represented by pitch, as in the previous algorithm” which pitches?

- We added the pitches used to the description of the original algorithm

I take it there is no scan line in this new algorithm version?

- That is correct (see above reply)

The writing is overly long and misses some methodological detail that should be included.

I would like the technical specifics on how the auditory properties of distance were made. What distances are being simulated by the subtractive synthesis (e.g. just within arm reaching distance, or did you simulate much further distances to exaggerate the changes in audio). Ecologically valid is perhaps overselling it unless you are replicating actual distances very precisely. To me it's more ecologically inspired mappings. How were the words made, are these synthetized voices or verbal recordings, we should not need to look up the other paper to know.

- We added the filter parameters used in the subsection describing the algorithm, as well as the details for the pitch modulation.

Regarding the description of the filter as ecological - this is a very important point. As you stated, it would be most accurate to describe it as ecologically inspired, as we've initially done in the abstract. We added the warranted clarification to the algorithm's description, and reviewed the manuscript to ensure no further misunderstanding may arise. As mentioned above, we also added to the discussion more details regarding further development of this implementation.

The baseline stage should be a separate paragraph since this is an important comparison made in the paper.

- We have separated this stage into a new paragraph.

Baseline only has 15 trials while the test has 90 trials. This should be mentioned as a limitation as there could be way more variability in the baseline results.

- We have mentioned this as a limitation in the relevant section of the discussion.

## Results

Try to be consistent in the naming of the conditions - e.g. Figure 2 is called the ‘reversed condition’ rather than interchanged. I think swapped is a better descriptor than interchanged or reversed.

- We have corrected this, and apologize for the previously confusing annotation.

Figure 4 should have a visual key on the meaning of different line colors and dotted lines.

- We have added these, and adapted the colors and patterns used to make the graphs more comprehensible.

If pitch appears to have a strong influence on whether the subject is correct or not, its important to note which pitches are used to help explain why that might be the case. Also subjects ability to discriminate pitch or their musical background would help elucidate these findings.

- As requested also for the methods section, we have added the pitches used to the relevant section.
  Regarding the subjects' musical background or auditory performance, this would indeed be relevant. We have added this to the Limitations and future directions section.

Figure 5 - there is a lot of dead space in these diagrams, which could be filled with explicit mentions of what audio is being heard in these - e.g. “High pitch, Low synthesis”

- We have now used the space for logos indicating the audio manipulation performed for each condition in each case. We used the same coding also to enhance Figure 2 (previously Figure 1) and the newly added spectrograms (Figure 1).

Ultimately I still don’t know the pitches or exact synthesis (i.e. what frequencies are being band pass filtered) are used in any of these conditions. If the vocal pitch is being altered, do low and high vocals sound like different genders? A spectrogram of these different audio mixes would be good to see, for example, the same word, altered in pitch and subtractive synthesis.

- We have added the details as requested in the Methods section, as well as added the spectrogram which we feel is indeed a very helpful addition for illustrating the auditory features.

So far it appears that the audio used, rather than what it represented is the key factor determining their success rates (after training). This might be expected if the key influencing factor is auditory discrimination rather than what it represents visually/spatially.

- This observation is in line with our conclusions following the results, as we discuss under the "TopoLanguageDepth implementation of depth representation SSD" subsection.

Figure 6 should have a title for each graph. Reminders on what each Z and Y axis represents (pitch, synthesis) would be appreciated are important when things are constantly switching back and forth. You could have patterning/outlines indicate whether the subject is hearing pitch changes or synthesis changes.

- We have added titles, as well as logos to indicate the audio manipulation performed for each condition in each case using the same visual system adopted throughout the paper.

Please illustrate significant comparisons on the graphs themselves. Please use colors to help separate the regular and reversed conditions so it is clear what everything related to visually. E.g. all regular data could be various shades of blue, while all reversed could be various shades of orange. Baseline can be lighter shades, and test can be darker shades (like you are currently doing). It’s probably better to have the condition (regular, reversed) stated first and the time (baseline, test) stated second on the graphs.

- We have now indicated significant comparisons on the graphs.
  Also in accordance with the previous comments, we have adopted a more consistent and coherent visual language to the different figures.

Everything so far has been baseline then test, and now in Table 3 its reversed? Please keep baseline than test consistent.

- We have fixed this.

Figure 7 - “axix” in title. If the diagrams are flipped, for the 2 diagrams on the right, shouldn’t the axes read columns (Y-axis) and rows (X-axis)? Because don’t the columns and rows refer to the stimuli subjects are interacting with and you’ve just ‘flipped’ the diagram, which means you should have also flipped the axes? Color axis is not named.

- We have corrected this both in figure 7 and in Figure 5.

## Discussion

How will X, Y, and Z be combined?

- We have added a more detailed description and further possibilities in the future directions section.

“Ecological principles” is still a very vague way of describing a band-pass filter.

- Following the previous comments we have made adjustments in referring to the ecologically inspired algorithm and also explained the rationale and details of the filter used more clearly. As such, we believe that the intention of applying ecological principles for designing the sensory substitution mapping should now be clearer.

This study explores depth perception, but also verticality, there is just as much evidence on verticality as there is depth.

- As detailed in the comment regarding this issue in the introduction, we have now explicitly mentioned verticality through the paper, and detailed how it was used to explore the main themes of this study.

In order to discuss when sensory substitution is intuitive, reference to the following paper would help a lot in knowing what has been explored previously

Stiles, N. R., & Shimojo, S. (2015). Auditory sensory substitution is intuitive and automatic with texture stimuli. Scientific reports, 5(1), 15628

- This is indeed a relevant and important reference. We have added it to the introduction to better contextualize the present study.

“indicating a high effectiveness for the training, and a high general learnability for these correspondences.” Are we considering the reversed condition also correspondences?

- It was indeed intended as a correspondence in the sense that a signal in one modality corresponds with one in another modality. We replaced this instance with "mapping" to avoid any unnecessary confusion.

If pitch works well with both elevation and proximity, there are a few papers on this as well as the following reference by Eitan:

Eitan, Z., Ornoy, E., & Granot, R. Y. (2012). Listening in the dark: Congenital and early blindness and cross-domain mappings in music. Psychomusicology: Music, Mind, and Brain, 22(1), 33.

- This is indeed very interesting. We have added this to our discussion with regards to how the visually impaired and blind perceive the pitch mapping

“steep learning curve with training times averaging under 15 minutes overall” Steep learning curve can also be interpreted as ‘very difficult to learn’, which is not the case here, so alternative phrasing may help clarity.

- We have now used alternate phrasing to clarify this as suggested.

“The significant differences in success rates in the original condition compared to the

interchanged one at baseline indicate that the chosen mapping was effective in building upon existing crossmodal correspondences and ecological cues.” This sentence is a good example of a general tendency throughout the writing, to use more words than required, when fewer can be easier to read and clearer - e.g. “At baseline, the original mapping significantly outperformed the reverse mapping, indicating the utility of crossmodal correspondences and ecological cues.”

- We have fixed this specific instance, and thoroughly edited the manuscript for conciseness and clarity more generally.

“It is interesting to note that two blind participants were the only two to report that they

perceived the interchanged condition to be intuitive.”

This is reminiscent of a finding in this abstract that the first presented mapping is the most intuitive for some blind subjects:

Hamilton-Fletcher, G., Pieniak, M., Stefanczyk, M., Chan, K., & Oleszkiewicz, A. (2020). Visual Experience influences associations between Pitch and Distance, but not Pitch and Height. Journal of Vision, 20(11), 1316-1316.

- This is indeed very relevant, we have incorporated the findings of this abstract to the manuscript.

Not all paragraphs are relevant to the specific research being conducted here, and instead feel like general writing about a topic that is unnecessary. For example lines 519-527. This is about how vision is stronger than audition for 3D discrimination, but so what? What relevance does this have for your specific questions, or sensory substitution in general? How does it influence SSD design?

- We have taken this into consideration and deleted this paragraph to improve the conciseness of the discussion.

There is a big difference between congenitally blind and late blind that is not commented on here, where it has an impact on their existing spatial abilities.

- This is a good point, we have now commented on this and added relevant references.

“Overall, it has been suggested that blind individuals have inferior spatial capabilities or perceptual abilities than the sighted.” This is way too broad a statement when the reference is about inferring visual perspective cues from sensory substitution. Monica Gori’s work on how visual impairments alter the perception of distance is far more suitable for a paragraph on general spatial hearing skills.

- We have now rephrased this statement to better reflect work such as that of Monica Gori, we have also added reference here to Monica Gori’s work, specifically her highly cited and relevant papers: “Impairment of auditory spatial localization in congenitally blind human subjects”, “Auditory and proprioceptive spatial impairments in blind children and adults”, and “Early visual deprivation severely compromises the auditory sense of space in congenitally blind children.”

Line 537 - Some of the language here is way too total for what is sometimes a minor difference in spatial discrimination skills between sighted and blind (early/late) individuals.

- We have rephrased this.

Don’t start a paragraph with ‘and yet’

- This has been corrected.

“This is unlike other visual-to-auditory sensory substitution devices that use arbitrary mappings of visual information to sounds.” Are they arbitrary? Are you considering cross-modal correspondences arbitrary? Most SSDs tend to use some cross-modal correspondences.

- As we have now better clarified our intention and mentioned the use of cross modal correspondences in existing SSDs already in the introduction, as suggested and detailed above, we have deleted this sentence.

The subject quotes sections could be heavily reduced, just to pick out the super relevant points.

- The subject quotes are the survey findings. As they are mainly qualitative and subjective, we include them in the discussion (as opposed to the results) and aim to present a transparent and unbiased representation of the responses. Following the comment, we reduced and shortened unnecessarily long or repetitive descriptions.

# Reviewer #2:

## General assessment

This is an interesting manuscript that explored the TopoLanguageDepth sensory substitution method for spatial perception, particularly depth perception, via a combination of auditory cues: naming objects and representing their elevation and depth. The authors explored the role of inherent and learned cues in depth perception as well as training of sensory mappings by perceptual learning through audition.

It is a proof of concept that included participants with normal vision (n=33), visually impaired (n=2), and blind (n=5) participants.

It is a well written manuscript with minor typos.However, I have some suggestions to improve the manuscript.

- Thank you for your positive assessment of this manuscript. We have now addressed your suggestions as detailed below and indicated the corresponding changes made in the manuscript by tracking the changes in the document file. We believe this has indeed improved the manuscript.

## Abstract

There is a typo in “...by comparing it to an in interchanged…”

- Thank you for pointing this out, we have fixed the typo.

## Introduction

There are repetitive mentions of the goals of the study (Ln95, 99, 105, 171). I suggest integrating them only in the final paragraph of the Introduction.

- We have now consolidated these mentions of the goal of the study, to the final paragraphs of the introduction as requested.

Ln139 Please, clarify the acronym “SSD” in its first mention

- Thank you for pointing this out. We have done as suggested.

Ln153-161, I suggest to improve the transition from the previous sentences, related to sight, and this section focused on audition.

- We have added a sentence to improve the transition.

## Methods

Ln184 Please include units in descriptive statistics (ie. years/yr)

- Thank you for pointing this out. We have added these units.

Ln 192 Please, clarify the Participants section, particularly how and where they were recruited. Were the participants patients or students from Reichman University?

- We have now added information about the participant recruitment procedure in the Participants section.

Did you perform a sample size calculation?

What were the inclusion/exclusion criteria?

- We did not perform a sample size calculation, but rather relied on related previous studies, including ones performed with the previous system (for examples, see Levy-Tzedek et al., 2012; Netzer et al., 2021; Ueda et al., 2019; Yizhar et al., 2021).
  The exclusion criteria were self-reported lack of neurological or hearing disorders and did not include hearing tests or other screenings. We have addressed both of these limitations in the relevant section of the discussion as also suggested in the comment below.

Ln193 I suggest to include the experiment conditions section, and clarify the groups (“conditions”; original and interchanged?) as well as how the visually impaired and blind participants were allocated to those groups.

- We have added this information as requested.

Ln196-202 I suggest that this information be removed or included in the Introduction so that this section is focused on the modifications from the original algorithm

- We have added to the introduction a high-level explanation of the original algorithm, and added in this section only the relevant information on the verticality representation and detailed technical parameters.

Ln206 Please justify naming “z-axis” when applying cues intended for depth perception when the representation is still in a 2D board. I suggest y1-axis and y2-axis to differentiate between conditions

- We now explained that the z-axis is relative to the user's space.
  As for the naming of the conditions, we wish to accentuate how the mapping of the interchanged condition is manipulated in relation to the original one, and as such prefer the explicit use of the terms. We added visual representation of the manipulation performed in each figure and color coded the conditions to help better explain the differentiation between conditions.

Ln260-262 There is no further mention of this interview, its structure, domains as well as the 9 questions. What was the purpose of this interview and where are the results? Please, clarify

- The interviews were conducted to gain qualitative insights into the quantitative results. Relevant findings were intertwined in the discussion to support or help clarify the findings. We now added the motivation to the interview description, as well as clarified that the quotes in the discussion originated in the interviews.
  Data, including the questions will be made available in a public repository upon acceptance as required.

Ln262 The authors should include a Statistical Analysis section, identifying the types of variables and their measurements, data processing, and the rationale for the statistical tests performed.

- We have added a statistical analysis section to the methods as recommended.

## Results

For all figures and tables, plese verify and homologate the nomenclature. Also, include more informative captions: units, groups, auditory cues, statistical tests performed,...

- Thank you for bringing this to our attention. We have now verified and maintained consistent nomenclature, and provided more informative captions.

Table 1

I recommend to include 95% confidence intervals when reporting the Means for a better interpretation of the uncertainty of the estimate

- We have included 95% confidence intervals when reporting the means.

Table 2

I suggesto to combine Table 2 and 4

- We have combined the tables as suggested.

Figure 2:

Are regular and reversed conditions referring to original and interchanged conditions? Please clarify and maintain a consistent nomenclature

- Yes they are, we have now made sure to maintain consistency, only using Original and Interchanged.

Please differentiate visually impaired and blind subjects in the Subjects column. That would help to identify where the visually impaired (n=2) and blind (n=5) participants were grouped, their success rate, individual and compared to the overall average.

- The visually impaired and blind participants are now differentiated

Figure 3.

Please clarify “Reversed” and “Regular”

- We have corrected this to “Original” and “Interchanged”.

Figure 4

I suggest that more contrasting colors be applied to differentiate between blind/visually impaired from sighted participants

- We have adjusted the color scheme and patterns used to make the differences more noticeable, and consistent with the color scheme adopted for representing the two conditions in the other figures in the paper.

Figure 5

Please include the rationale for the flipped diagram in the Methods - Data analysis or Statistical analysis section

It is not clear to me why was this performed, and why only for the interchanged condition instead of both conditions. In this regard, and if the figure does not changes, I suggest to position the flipped diagram below its corresponding panel (interchanged).

- The flipped diagrams were meant to enable an intuitive comparison of the separate sounds between the original and interchanged configurations, which is why we also preferred placing it either next to or under the original condition's results rather than diagonally. We better explained this rationale in the Results section, and added an indication of the audio produced in each cell to better clarify the diagram and its comprehension in the individual cell level.

How inherent and learned cues participate in depth perception?

- We now added in the relevant result a specific note on the intuitiveness of the mappings reliant on inherent correspondences compared to the mapping which required learning, as further discussed later in the discussion.

Please, clarify how spatial abilities of visually impaired and blind can converge with those of the sighted?

- We also added a specific note on this in the results, discussing the statistically similar results of the different groups. It is important to mention, following reviewer 1's comment on the abstract, that the conclusion on convergence of performance in this case does not imply general convergence of performance but rather supports the convergence view as described in the discussion.

Please verify that the supplementary material be mentioned in the manuscript.

- Following this comment and a re-consideration of the value of these heat maps, we decided to remove the supplementary material.

Ln326 and 360 Please clarify substractive synthesis for elevation instead of distance (Ln206)

- In these instances, we are speaking about the interchanged condition, in which the subtractive synthesis was used to represent elevation, and pitch represented depth. We believe the logos added to the figures indicating the audio features applied in each condition would assist in preventing any further confusion.

## Discussion

Ln352 Along the manuscript, there is a vast diversity of mentions related to the “ecological” domain such as “ecological principles”, “ecological mapping”, “ecological information”, “ecological behavior”, “ecological considerations”, “ecological cues”, “ecologically inspired”, “ecological representation” of auditory properties. Please clarify that domain since its first mention in the Introduction section (what is meant by "ecological" in this context?), and discuss the related results accordingly (ie. how well the algorithm captures and represents auditory cues in a manner consistent with natural depth or distance perception?).

- We have now further clarified this throughout the manuscript, specifically referring to the algorithm as ecologically inspired, and better explain the implementation and considerations both in the introduction and method sections as discussed also in the previous responses to related comments by Reviewer 1 and the editor.

Ln564 Were the one or two participants that reached 100% accuracy in the y-axis or z-axis, respectively, the participants that found intuitive the interchanged condition?

- Thank you for inquiring about this. Indeed they were. We have added this to the discussion section.

Ln597 I suggest to highlight the small sample size, in general, and particularly for blind and visually impaired participants. In addition, as this was a proof of concept and a sample size calculation was not performed, the findings cannot be generalized. Additional limitations might include the lack of inclusion/exclusion criteria that might impact your results such as hearing functions (ie. audiometry or sound localization tests)

- We have highlighted these limitations more clearly and explicitly in the limitations section of the discussion.

## Conclusions

Please, include this section

- We have now included a conclusions section as requested.
